# Development of an immunochromatography strip test based on truncated nucleocapsid antigens of three representative hantaviruses

**DOI:** 10.1186/1743-422X-11-87

**Published:** 2014-05-14

**Authors:** Takako Amada, Kumiko Yoshimatsu, Takaaki Koma, Kenta Shimizu, Chandika D Gamage, Kanae Shiokawa, Sanae Nishio, Clas Ahlm, Jiro Arikawa

**Affiliations:** 1 Department of Microbiology, Graduate School of Medicine, Hokkaido University, Kita 15, Nishi 7, Kita ku, Sapporo 060-8683, Japan; 2 Division of Infectious Diseases, Department of Clinical Microbiology, Umeå University, Umeå S-901 85, Sweden

**Keywords:** Immunochromatography strip test, Hemorrhagic fever with renal syndrome (HFRS), Nephropathia epidemica (NE), Hantavirus pulmonary syndrome (HPS)

## Abstract

**Background:**

Hantaviruses are causative agents of hemorrhagic fever with renal syndrome (HFRS) and nephropathia epidemica (NE) in the Old World and hantavirus pulmonary syndrome (HPS) in the New World. There is a need for time-saving diagnostic methods. In the present study, recombinant N antigens were used as antigens in an immunochromatography strip (ICG) test to detect specific IgG antibodies.

**Methods:**

The N-terminal 103 amino acids (aa) of Hantaan virus (HTNV), Puumala virus (PUUV) and Andes virus (ANDV) nucleocapsid (N) protein were expressed in *E. coli* as representative antigens of three groups (HFRS, NE and HPS-causing viruses) of hantavirus. Five different types of ICG test strips, one antigen line on one strip for each of the three selected hantaviruses (HTNV, PUUV and ANDV), three antigen lines on one strip and a mixed antigen line on one strip, were developed and sensitivities were compared.

**Results:**

A total of 87 convalescent-phase patient sera, including sera from 35 HFRS patients, 36 NE patients and 16 HPS patients, and 25 sera from healthy seronegative people as negative controls were used to evaluate the ICG test. Sensitivities of the three-line strip and mixed-line strip were similar to those of the single antigen strip (97.2 to 100%). On the other hand, all of the ICG test strips showed high specificities to healthy donors.

**Conclusion:**

These results indicated that the ICG test with the three representative antigens is an effective serodiagnostic tool for screening and typing of hantavirus infection in humans.

## Background

Hantaviruses belong to the genus *Hantavirus* of the family *Bunyaviridae*. Hantavirus virions contain three segmented negative-sense RNAs designated S, M, L that encode a nucleocapsid (N) protein, enveloped glycoproteins (Gn and Gc), and an RNA-dependent RNA polymerase (L protein), respectively [1]. Viruses in the genus *Hantavirus* contain causative agents of two important rodent-borne febrile zoonoses, hemorrhagic fever with renal syndrome (HFRS) in the Old World and hantavirus pulmonary syndrome (HPS) in the New World [2].

So far, 24 virus species that represent serotypes and genotypes have been registered within the genus *Hantavirus* by the International Committee on Taxonomy of Viruses [3]. Four of those virus species, Hantaan virus (HTNV), Seoul virus (SEOV), Dobrava-Belgrade virus (DOBV), and Puumala virus (PUUV), are known to cause HFRS. The milder type of HFRS in Northern Europe, caused by PUUV infection, was historically called nephropathia epidemica (NE). Sin Nombre virus (SNV), Andes virus (ANDV), Laguna Negra virus (LANV), and many other related viruses are known as causative agents of HPS [4]. There is a close association between the virus species and their rodent hosts, probably due to the co-evolution of rodent hosts and viruses for many thousands of years [5]. As a consequence, endemic areas of HFRS and HPS depend on the rodent habitat. However, imported cases of HFRS and HPS between different countries and continents have been reported [6-8]. Furthermore, more than one hantavirus species is present in some regions, and the severity of disease differs depending on the virus [9]. In addition, clinical diagnoses of HFRS and HPS patients are not effective in some cases as they represent mixed-syndrome between HFRS and HPS [10,11]. Therefore, screening and typing of hantavirus infections provides important epidemiologic information.

Hantaviruses can be divided into antigenically distinguished species by neutralization tests, which show the antigenic differences of Gn and/or Gc proteins. While, it has been reported that hantavirus species can be divided into three antigenic groups based on antigenic cross-reactivity mainly at the N terminal end of N protein: group I (HTNV, SEOV, and DOBV, which are derived from Murinae rodents), group II (PUUV, and nonpathogenic vole-borne hantaviruses, Tula virus, Prospect Hill virus, and others, those are derived from Microtinae rodents) and group III (SNV, ANDV and related New World hantaviruses derived from Sigmodontinae and Neotominae rodents) [12]. Therefore, three kinds of antigens from each of the three antigenic groups I, II, and III are necessary for screening of all of the rodent-borne hantavirus infections.

We previously developed an *Escherichia coli* (*E. coli*) and baculovirus-expressed recombinant N protein for screening and serotyping patient samples in an enzyme-linked immunoassay (ELISA) for HFRS and HPS viruses [12,13]. Herein, we describe a pilot study to assess a new rapid immunochromatography (ICG) test with recombinant antigens using *E. coli-*expressed common antigenic sites located in the N terminal end of N protein for detection of specific IgG antibodies in acute and convalescent samples of patients with clinical infection caused by various hantavirus species. In addition, the ICG test was evaluated for the ability to discriminate among the three main hantavirus groups.

## Results

### ICG test lines of different ICG strips

**Figure 1:**
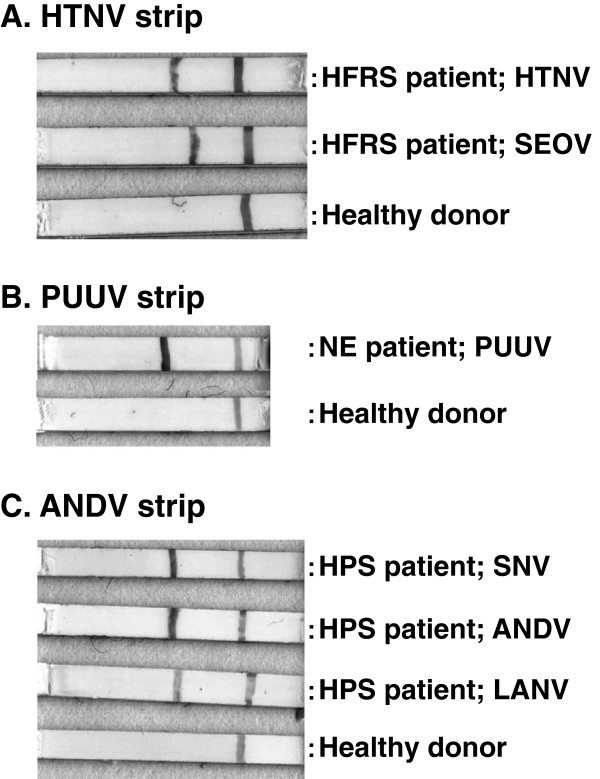
**ICG test lines of the HTNV, PUUV and ANDV strips. A**: Test lines of the HTNV strip with sera from HFRS patients infected with HTNV and SEOV. **B**: Test lines of the PUUV strips with sera from an NE patient infected with PUUV and a seronegative sera from healthy donor. **C**: Test lines of the ANDV strip with sera from HPS patients infected with SNV, ANDV and LANV.

**Figure 2:**
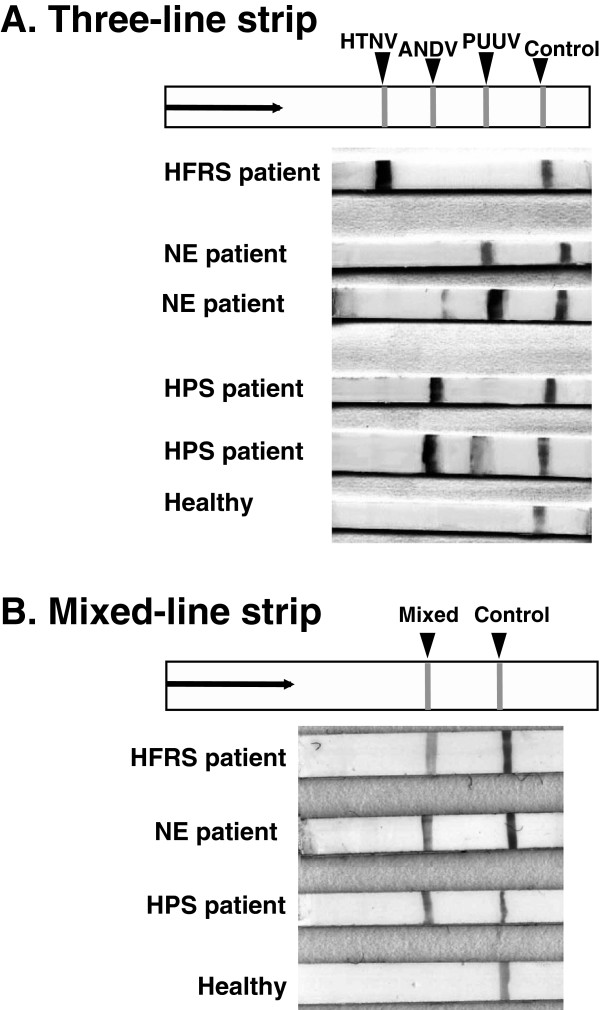
**ICG test lines of the three-line strip and mixed-line strip. A**: Test lines of the three-line strip with sera from HFRS, NE and HPS patients and a seronegative healthy donor. **B**: Test lines of the mixed-line strip with sera from sera from HFRS, NE and HPS patients and a seronegative healthy donor.

The HTNV, PUUV and ANDV strips for IgG antibody detection, which had one antigen line on one strip, showed clear lines against homologous patient sera infected with group I (HTNV and SEOV), group II (PUUV) and group III (SNV, ANDV and LANV) viruses (Figure 1). On the other hand, no line was observed with seronegative sera from healthy donors. In some cases, the NE and HPS patient sera showed cross-reactions to heterologous ANDV or PUUV strips with lower intensity lines, but no cross-reaction was observed in HFRS patient sera to ANDV or PUUV strips (Figure 2A). Similarly, the HTNV strip showed no test line with NE and HPS patient sera (data not shown). As can be seen in Figure 2B, the mixed-line strip showed a clear test line and control line to HFRS, NE and HPS patient sera.

### Comparison of the results of ICG tests with patient sera

A total of 149 patient sera were examined for their IgG antibodies to hantavirus by using 5 different ICG strips (Table 1). Among them, 87 convalescent-phase patients sera were used for evaluation of the ICG test. The HTNV, PUUV and ANDV strip showed sensitivities to homologous sera of 100%, 97.2% and 100%, respectively. The three-line strip showed sensitivities to homologous sera of 100%, 100% and 100%, respectively. Mixed-line strip showed sensitivities to patient sera of 100%, 97.2% and 100%, respectively.

**Table 1:** Comparison of reactivities of HFRS, NE and HPS patient sera in ICG tests and ELISA

**Type of disease**	**Infected with**	**Phase**	**No. serum**	**ELISA**		**Single antigen-strip**			**Three-line strip**			**Mixed-line strip (+)**
				**IgG (+)**	**IgM (+)**	**HTNV (+)**	**PUUV (+)**	**ANDV (+)**	**HTNV (+)**	**PUUV (+)**	**ANDV (+)**	
HFRS	HTNV or SEOV	acute	21	19	17	21 (100%)	1 (4.7%)	1 (4.7%)	21 (100%)	1 (4.7%)	0 (0%)	20 (95%)
		conv.^a^	35	35	20	35 (100%)	1 (2.8%)	0 (0%)	35 (100%)	1 (2.8%)	0 (0%)	35 (100%)
NE	PUUV	acute	26	21	24	0 (0%)	24 (92.3%)	2 (7.7%)	1 (3.8%)	23 (88.5%)	10 (38.5%)	24 (92.3%)
		conv.	36	36	5	0 (0%)	35 (97.2%)	17 (47.2%)	0 (0%)	36 (100%)	7 (19.4%)	35 (97.2%)
HPS	SNV, ANDV or LANV	acute	12	7	4	0 (0%)	4 (33.3%)	12 (100%)	0 (0%)	3 (25%)	12 (100%)	12 (100%)
		conv.	16	16	3	0 (0%)	10 (62.5%)	16 (100%)	0 (0%)	8 (50%)	16 (100%)	16 (100%)
Seronegative healthy donar	-	-	25	0	0	0 (0%)	0 (0%)	0 (0%)	0 (0%)	0 (0%)	0 (0%)	0 (0%)

^a^Convalescent.

Twenty-one HFRS patient sera from China, 9 NE patient sera from Germany, 35 NE patient sera from Sweden, 11 HPS patient sera from U. S. A., and 17 HPS patient sera from Argentina were used in this study.

The HTNV, PUUV and ANDV strips showed high sensitivities to homologous sera with 100%, 92.3% and 100% in acute-phase, respectively. The three-line strip showed almost the same sensitivities: 100%, 88.5% and 100% in acute-phase sera, respectively. The mixed-line strip showed sensitivities of 95%, 92.3% and 100% in acute-phase sera, respectively.The PUUV strip was able to detect antibodies in 14/28 (50%) of the HPS patient sera (acute and convalescent) but in only 2/56 (3.6%) of the HFRS patient sera (acute and convalescent). Similarly, the ANDV strip was able to detect antibodies in 19/62 (30.6%) of the NE patient sera (acute and convalescent) but in none of the HFRS patient sera. The HTNV strip, on the other hand, showed no cross-reactivity to NE and HPS patient sera. The three-line strip showed the same cross-reactive patterns as those of the HTNV, PUUV and ANDV strips. In the three-line strip, all of the cross-reactive sera showed a weak band to the heterologous antigen compared to that with the homologous antigen, as shown in Figure 2.

### Comparison of sensitivities of the 5 different ICG strips with ELISA

Representative sera from SEOV, ANDV or PUUV-infected patients in convalescent phase were examined for antibody titers with the 5 different ICG strips and ELISA. The single antigen strips, including the HTNV strip, ANDV strip and PUUV strip, and the three-line strip showed the same titers as that of ELISA with homologous combinations. The mixed-line strip also showed the same titer as that of ELISA (data not shown). Therefore, detection limit of IgG antibody was almost the same as that of conventional ELISA.

## Discussion

In this study, N-terminal 103 aa of N protein of HTNV, PUUV and ANDV expressed in *E. coli* were successfully applied as antigens for an ICG test to detect hantavirus IgG antibody in patient sera.

It has been reported that hantavirus N protein contains immunodominant and cross-reactive epitopes, a characteristic that is common to HFRS-causing viruses such as HTNV, SEOV and DOBV (group I), NE-causing viruses such as PUUV (group II) and HPS-causing viruses such as SNV, ANDV, and LANV (group III), at about the N-terminal 100 aa region.

In this study, we demonstrated that N-terminal 103 aa of N protein of one virus from each group were useful antigens in the ICG test for a rapid and simple serological test to screen for hantavirus IgG antibodies in human sera. Five different types of ICG test strips were evaluated. Sensitivities were almost the same for all of the ICG strips and ELISA test. The three-line strip was considered to be the most effective as it covered all of the hantavirus infections as well as distinguishing its antigenic groups. Unrecognized cases of hantavirus infection might exist even in countries where HFRS or HPS prevalence has not been reported. To know the actual situation of hantavirus prevalence, serological surveillance against all of the serotypes of hantavirus is required. Particularly in Europe and Far Eastern Asian regions, both HFRS and NE-causing viruses are co-circulating [14,15]. For this purpose, the multiplex three-line strip ICG test is considered to be the most useful for a rapid and safe serological test as it covers all antigenic groups of human pathogenic hantaviruses. Furthermore, the ICG test was applicable to diluted whole blood instead of serum specimens [16]. This allows the use of the ICG test in field surveillance or in resource-limited settings where adequate laboratory equipment is not available. An ICG test to detect IgM antibody has been reported for diagnosis of NE [17,18]. Our ICG tests were developed for IgG antibody detection for the purpose of retrospective seroepidemiological study. Although the rates of detection of anti-hantavirus IgG antibody evaluated with convalescent phase patient sera were more than 97.2%. One convalescent-phase NE patient serum showed negative results in both single PUUV strip and mixed-line strip. This specimen showed low ELISA OD values and made the sensitivities of ICG tests for NE low (97.2%) comparing to HPS and HFRS (100%). Totally, three-line strip showed relatively better sensitivity than those of single-antigen and mixed-line strips by evaluation with convalescent.

On the other hand, antibody-positive rates in acute-phase sera (88.5 - 100%) were slightly lower than those in convalescent-phase sera. This might be due to the lower IgG antibody titer in acute-phase sera. Furthermore, 3 of the ICG-negative sera from NE acute patients (Table 1) were collected 4 and 5 days after onset of the disease. These results indicated the limitation of these ICG strips. All of the patient sera used in this study were derived from patient with confirmed hantavirus infection. Therefore, even if sera were negative in ELISA, they were considered as true positive. Although several sera from acute-phase patients were IgG-negative in ELISA, the results of sensitivities from acute-phase patients must be informative for the use of these ICG strips.

Protein A binds with high affinity to human IgG antibody molecule as well as to human IgM antibody molecule with moderate affinity. Therefore, our ICG test which utilized Protein A-labeled colloidal gold, able to detect both hantavirus IgG antibody and lesser extent IgM antibody. However, since the ICG test was judged by the detection of visualized accumulated colloidal gold particles, it is difficult to distinguish IgG and IgM detection. Therefore, the ICG test for specific to IgM antibody detection is necessary for detecting antibody from acute-phase patient sera and for differential diagnoses between acute infection and past infection in endemic countries.

The three-line strip developed in this study enables a homologous reaction and a hererologous cross-reaction to be easily distinguished by the intensity of lines. On the other hand, the mixed-line strip enables detection of hantavirus antibody in all of the three antigenic groups with high sensitivity.

## Conclusion

This applicability indicates that a multiplex ICG strip or the mixed-line strip or single antigen-strips were useful for serological survey of hantavirus infections among human. Depending on the situation, these strips were effective for survey. To test imported cases found in non-endemic countries, one strip have to screen for various antigens including similar infectious diseases, such as leptospirosis, severe fever with thrombocytopenia syndrome (SFTS) and other viral hemorrhagic fever. On the other hand, in endemic area and its neighbor, use of single antigen strip might be enough for screening. These ICG strips reported in this paper would be a useful tool for serological surveillance of hantavirus infection.

## Methods

### cDNAs

cDNAs containing coding information for N of ANDV [8], HTNV strain 76–118 [19], and PUUV strain CG1820 [20] were used for the preparation of truncated N antigens.

### Sera

A total of 56 serum specimens from 21 HFRS patients (12 pairs, 4 triads and 5 quartets, including acute-phase serum) were kindly provided by H. Wang of the Institute of Virology, Chinese Academy of Preventive Medicine, Beijing, China. These sera were confirmed to be positive for hantavirus infection by neutralization test as previously reported [21]. Because of the lack of precise records, the sera collected the first time were regarded as acute-phase sera and rest of them were regarded as convalescent-phase sera.

Nine NE patient sera, including 4 acute-phase sera and 5 convalescent-phase sera, were kindly provided by Dr. Rainer Ulrich, Friedrich-Loeffler Institut, Federal Research Institute for Animal Health, Institute for Novel and Emerging Infectious Diseases, Germany. These sera were confirmed to be antibody-positive to PUUV by a neutralization test [22,23]. A total of 17 sera, including 4 acute-phase and 13 convalescent-phase sera, were kindly provided by Dr. Ake Lundkvist, Swedish Institute for Infectious Disease Control, Sweden and were confirmed to be antibody-positive to PUUV as previously described [24]. In addition, a total of 18 paired (acute and convalescence-phase) sera obtained from hospitalized NE patients in northern Sweden were included. They were confirmed to be infected with PUUV by an indirect immunofluorescence assay (IFA).

Eleven serum specimens from HPS patients infected with SNV in the United States were kindly supplied by Brian Hjelle of the University of New Mexico Health Sciences Center. Eleven serum specimens from HPS patients infected with ANDV and six serum specimens from HPS patients infected with LANV, which is the most antigenically related to ANDV and is co-circulating in the area where ANDV is prevalent, were obtained from Dr. Delia Enria, Instituto Nacional de Enfermedades Vigrales Humanas and Dr. Julio I. Maiztegui, Pergamino, Argentina. Twenty-five sera from healthy donors that were confirmed to be hantavirus antibody-negative were used as a negative control group [24]. This study was approved by the ethics committee of Hokkaido University Graduate School of Medicine.

### Preparation of recombinant antigens

Hantavirus N protein possesses an immunodominant antigenic region within the N-terminus of about 100 aa [25-27]. In this study, N-terminal 103 aa of N protein of HTNV (HS103), PUUV (PUU103) and ANDV (AND103) were selected as representative antigens of the three antigenic groups. HS103 and PUU103 were expressed as an NUS and 6 X histidine -tagged recombinant protein using the pET43.1 plasmid vector (Novagen, Madison, WI, USA) and *E. coli* host strain BL21 (Novagen pET System Manual 10th ed.) as described previously [16,23]. The coding region of AND103 was amplified using primers ANDVSATG-speI (5′-aaa*actagt*atgagcaacctccaagaa-3′) and ANDVS103stop-xho1 (5′ - aat*ctcgag*ttaatccaggacatttccata -3′) by a previously described procedure [12]. A TAA stop codon, indicated by an underline, was added to terminate recombinant protein translation. Additional target sequences for the restriction enzymes SpeI and XhoI are indicated by italics. Fragments were cloned into the SpeI/XhoI restriction site of the pET43.1 vector in-frame with the NUS and histidine (his)-tag, and the resultant plasmids were introduced into *E. coli* host BL21 (DE3). HTN103, PUU103 and AND103 were purified using HisTrap HP columns (Amersham, Piscataway, NJ, USA) according to the manufacturer’s instructions. Purified antigens were evaluated by SDS- PAGE analysis followed by protein staining with Simply blue (Invitrogen, Carlsbad, CA, USA, data not shown).

### Enzyme-linked immunosorbent assay (ELISA)

#### (i) IgG ELISA

Ninety-six-well plates were first coated with 1 μg/ml of HS103, PUU103 or AND103 in PBS for 1 h at 37°C as a capture antigen. After being washed three times with PBS containing 0.05% Tween 20 (PBS-T), the wells were blocked with PBS containing 3% bovine serum albumin (BSA) for 1 h at 37°C. After blocking, human sera at 1:200 dilution with ELISA buffer (PBS containing 0.5% BSA and 0.05% Tween 20) were added to the wells. After incubation for 1 h at 37°C, the wells were washed three times with PBS-T. Bound antibody was detected with horseradish peroxidase (HRP)-labeled goat anti-human IgG antibody (Invitrogen™) for 1 h at 37°C. After being washed as above, color reactions were performed with o-phenylenediamine dihydrochloride (OPD) (Sigma-Aldrich, St. Louis, MO) and allowed to develop for 10 min. Absorbance was measured at 450 nm by using a SpectraMax 340 microplate spectrophotometer (Molecular Device, Sunnyvale, CA). The optical density (OD) of each test sample was subtracted from the OD value of the control antigen, which was a purified NUS-tag protein alone, run on the same plate. Samples with OD values of less than 0.2 were considered negative for IgG antibody.

#### (ii) IgM ELISA

IgM ELISA was carried out as previously described [28]. Briefly, ninety-six-well plates were coated overnight at 4°C with goat anti-human-μ-chain antibody (Cappel, Aurora, OH). After washing and blocking as described above, patient sera at 1:200 dilution were added to capture IgM antibodies in sera. Then a recombinant N protein of HTNV, PUUV or ANDV, prepared by using a baculovirus vector as described previously [12,13], was added. To detect bound antigens, biotinylated MAb clone E5/G6 against N protein of HTNV [29,30] was added. After reaction with streptavidin-HRP conjugate (Prozyme), the color was developed with TMB (3,3′,5, 5′-tetramethylbenzidine) for 15 min, and development was stopped with 0.5 M sulfuric acid. Absorbance was measured at 450 nm by using a SpectraMax 340 microplate spectrophotometer (Molecular Device). Samples with OD values of less than 0.2 were considered negative for IgM antibody. Reagena Poc Puumala® was used for detection of IgM antibody against PUUV in NE patient sera at the Department of Clinical Virology, Umeå University Hospital [17,18].

### Preparation of an ICG strip

#### (i) Preparation of a conjugate pad

A glass-fiber conjugate pad (5 mm × 300 mm, GFCP103000, MILLIPORE) was soaked in 0.5% casein in 20 mM PBS (pH 7.0) for 20 min. The pad was washed with 3.0% sucrose PBS and dried in air overnight. Protein A-conjugated colloidal gold (Protein A-Colloidal Gold conjugate, EY Laboratories, CA., USA) was put onto the pad at 500 μl for both ICG strips “Single-antigen strip” and “Mixed-line strip”, and at 1000 μl for the “three-line strip” described as below. The pad was dried in air overnight and kept at room temperature.

#### (ii) Preparation of a nitrocellulose membrane

**Figure 3:**
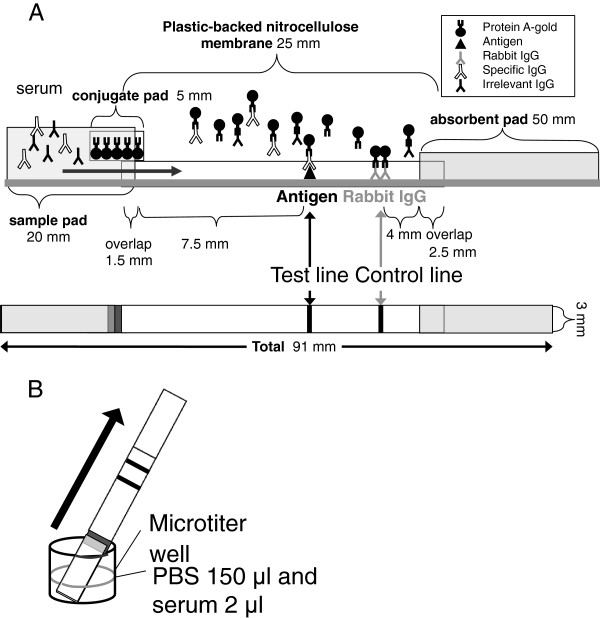
**Detection method and scheme of ICG strip. A**: Structure of ICG strip. N-terminal 103 amino acids of N protein and rabbit IgG were placed at the test line and control line, respectively. The ICG strip consisted of 4 membrane pads: sample pad, conjugate pad, nitrocellulose membrane and absorbent pad. Protein A-colloidal gold conjugate was kept in the conjugate pad. **B**: Detection method. Two μl of serum was diluted with 150 μl of PBS and then placed in a microtiter well. The strip was dipped in the solution. After standing still for 15 min, the test line and/or control line appeared.

HS103, PUU103 and AND103 antigens prepared as described above and rabbit IgG (Vector Laboratories, Burlingame, USA) were diluted with 0.05% casein sodium in 2.5 mM PBS (pH 7.0) to adjust the concentrations to 1 mg/ml and 0.15 mg/ml, respectively. Then they were immobilized on a nitrocellulose membrane (Hi-Flow Plus 240 Membrane Card, 60 mm × 300 mm, Millipore) by drawing a thin line with a pen (Copic Sketch Spare Nib, Too Co. Ltd, Japan) at the test line position (antigen) and control line position (rabbit IgG). ICG strips with HS103, PUU103 and AND103 antigen lines are named HTNV strip, PUUV strip and ANDV strip, respectively, in the text. The ICG strip with three antigens on separated lines in one strip is named a three-line strip, and the ICG strip with a single antigen line of three mixed antigens is named a Mixed-line strip in the text. After the membrane had been dried at room temperature for 15 min, it was soaked in 20 mM PBS with 0.5% casein (pH 7.0) for 20 min to block the unsaturated area. Then the membrane was washed with 3.0% sucrose in DDW and dried overnight at room temperature. Finally, a sample pad (20 mm × 300 mm, Chromatography Paper 3MM Chr, Whatman), conjugate pad, nitrocellulose membrane and absorbent pad (50 mm × 300 mm, Cellulose fiber sample pad CFSP203000, Millipore) were assembled on a membrane card (Millipore). Then the combined membranes were cut into 3-mm-wide strips by using a paper cutter (Kokuyo, PT62, Japan). The structure of the ICG strip is shown in Figure 3A. The ICG strip was stored at room temperature in a dry and dark box until use.

#### (iii) Procedure for the ICG test

Serum dilution was prepared by mixing 2 μl of serum with 150 μl of PBS in a microplate well, and an ICG strip was directly put into the well (Figure 3B). After standing still for 15 min, the ICG line was examined by the naked eye. A serum sample that showed both test and control lines was regarded as positive, and a serum sample that showed only the control line was regarded as negative.

## Abbreviations

ICG: Immunochromatography; HFRS: Hemorrhagic fever with renal syndrome; NE: Nephropathia epidemica; HPS: Hantavirus pulmonary syndrome; HTNV: Hantaan virus; SEOV: Seoul virus; DOBV: Dobrava-Belgrade virus; PUUV: Puumala virus; SNV: Sin Nombre virus; ANDV: Andes virus; LANV: Laguna Negra virus.

## Competing interests

Authors declare that they have no competitng interests.

## Authors’ contributions

TA, Preparation of ICG strip and recombinant AND103 antigen and evaluation of them by using patient sera. KY, Preparation of recombinant antigen of HS103 and PUU103, characterization of HFRS patient sera. TK, Characterization of HPS patient sera. KS IgM and IgG ELISA. CDG, Characterization of HFRS patient sera. KS, Preparation of ICG strip. SN, Preparation of ICG strip. CA, collection and characterization of NE patient sera. JA, superintendence the whole research. All authors read and approved the final manuscript.
